# Type 2 diabetes mellitus caused by Gitelman syndrome-related hypokalemia: A case report

**DOI:** 10.1097/MD.0000000000021123

**Published:** 2020-07-17

**Authors:** Guangyu He, Xiaokun Gang, Zhonghua Sun, Ping Wang, Guixia Wang, Weiying Guo

**Affiliations:** a Department of Endocrinology and Metabolism; b Department of Otolaryngology-Head and Neck Surgery, The First Hospital of Jilin University, Changchun, Jilin, P.R. China.

**Keywords:** case report, diabetes mellitus, Gitelman syndrome, hypokalemia, *SLC12A3* gene

## Abstract

**Introduction:**

Gitelman syndrome (GS) is an autosomal-recessive disease caused by *SLC12A3* gene mutations. It is characterized by hypokalemic metabolic alkalosis in combination with hypomagnesemia and hypocalciuria. Recently, patients with GS are found at an increased risk for developing type 2 diabetes mellitus (T2DM). However, diagnosis of hyperglycemia in GS patients has not been thoroughly investigated, and family studies on *SLC12A3* mutations and glucose metabolism are rare. Whether treatment including potassium and magnesium supplements, and spironolactone can ameliorate impaired glucose tolerance in GS patients, also needs to be investigated.

**Patient concerns:**

We examined a 55-year-old Chinese male with intermittent fatigue and persistent hypokalemia for 17 years.

**Diagnoses:**

Based on the results of the clinical data, including electrolytes, oral glucose tolerance test (OGTT), and genetic analysis of the *SLC12A3* gene, GS and T2DM were newly diagnosed in the patient. Two mutations of the *SLC12A3* gene were found in the patient, one was a missense mutation p.N359K in exon 8, and the other was a novel insert mutation p.I262delinsIIGVVSV in exon 6. *SLC12A3* genetic analysis and OGTT of 9 other family members within 3 generations were also performed. Older brother, youngest sister, and son of the patient carried the p.N359K mutation in exon 8. The older brother and the youngest sister were diagnosed with T2DM and impaired glucose tolerance by OGTT, respectively.

**Interventions:**

The patient was prescribed potassium and magnesium (potassium magnesium aspartate, potassium chloride) oral supplements and spironolactone. The patient was also suggested to maintain a high potassium diet. Acarbose was used to maintain the blood glucose levels.

**Outcomes:**

The electrolyte imbalance including hypokalemia and hypomagnesemia, and hyperglycemia were improved with a remission of the clinical manifestations.

**Conclusion:**

GS is one of the causes for manifestation of hypokalemia. *SLC12A3* genetic analysis plays an important role in diagnosis of GS. Chinese male GS patients characterized with heterozygous *SLC12A3* mutation should be careful toward occurrence of T2DM. Moreover, the patients with only 1 *SLC12A3* mutant allele should pay regular attention to blood potassium and glucose levels. GS treatment with potassium and magnesium supplements, and spironolactone can improve impaired glucose metabolism.

## 1 Introduction

Gitelman syndrome (GS) is a rare autosomal-recessive renal tubular disease; its prevalence among the general population is 1% to 3%.^[1,2]^ The main clinical manifestations of GS are hypokalemic metabolic alkalosis with hypomagnesemia and hypocalciuria, hyperreninemia, and hyperaldosteronism.^[3,4]^ GS is diagnosed in a patient based on the following criteria: documented chronic hypokalemia (<3.5 mmol/L) concomitant with inappropriate renal potassium wasting (spot urine, potassium creatinine ratio >2.0 mmol/mmol), absence of potassium-lowering drugs; metabolic alkalosis; hypomagnesemia (<0.7 mmol/L), inappropriate renal magnesium wasting (fractional excretion of magnesium >4%); hypocalciuria (spot urine, calcium–creatinine ratio <0.2 mmol/mmol) in adults, normal calcium–creatinine ratio is different in children; high renin (activity or plasma levels); fractional excretion of chloride >0.5%; normal or low blood pressure; normal renal ultrasound with absence of nephrocalcinosis or renal abnormalities.^[5]^ Symptoms or features that do not support GS diagnosis are as follows: chronic use of diuretics or laxatives; family history of renal malformations or any kidney disease transmitted as a dominant trait; presence of a renal malformation (e.g., unilateral kidneys, polycystic kidneys, etc.); history of polyhydramnios or hyperechogenic fetal kidneys; presentation before the age of 3 years; lack of hypokalemia or inconsistent hypokalemia in the absence of substitutive therapy; long history of hypertension; manifestation of increased extracellular fluid volume. However, the presence of arterial hypertension does not exclude the diagnosis of GS in adults.^[5]^ Confirmation of clinically suspected GS diagnosis rests on genetic testing, which should be conducted for all subjects. The diagnosis of GS is proven by identification of biallelic inactivating mutations of the *SLC12A3* gene, which encodes for the thiazide-sensitive sodium-chloride cotransporter in the distal convoluted tubules.^[6,7]^ To date, more than 180 mutations of the *SLC12A3* gene, both exonic and intronic, have been reported in patients with GS.^[3,8]^

Recently, patients with GS have been reported to be at an increased risk for developing type 2 diabetes mellitus (T2DM). However, hyperglycemia has not been thoroughly investigated in patients with GS. Studies on glucose metabolism in GS patients and their families are also rare. Whether GS treatments including supplements of potassium and magnesium, and spironolactone can ameliorate impaired glucose tolerance in GS patients also needs to be investigated.

In the present study, a patient with persistent hypokalemia was diagnosed with GS and T2DM by gene *SLC12A3* analysis and oral glucose tolerance test (OGTT), respectively. Analyses of the *SLC12A3* gene and OGTT of 9 family members within 3 generations were also carried out to determine the relationship between the *SLC12A3* mutations and impaired glucose metabolism. The mechanisms that could cause T2DM in GS patients and clinical features of GS patients with T2DM have also been discussed in this study.

## 2 Case report

A 55-year-old Chinese male patient with intermittent fatigue was examined in this study. The patient's symptoms started 17 years ago with fatigue without an obvious cause, followed by occasional limb weakness and stiffness; this was accompanied with palpitations. Hypokalemia was diagnosed by testing blood electrolyte levels. The patient took potassium supplements (potassium citrate) irregularly; however, this did not normalize the patient's potassium levels. Two weeks before the patient was admitted to the hospital, the above-mentioned symptoms were aggravated after drinking juice. Blood pressure and body mass index (BMI) of the patient were 104/76 mm Hg and 20.76 kg/m^2^, respectively. Cardiopulmonary, abdominal, and neurological examinations showed no abnormalities. The patient's mother had been previously diagnosed with hypokalemia and died 10 years ago. His parents were nonconsanguineous, and none of his siblings had hypokalemia.

The results of the main laboratory tests of the patient have been summarized in Table 1. The laboratory tests showed hypokalemia, hypomagnesemia, and mild hypochloremia. Urine analysis revealed inappropriate kaliuresis, abundant natriuresis, abundant chlorosis, and hypocalciuria. The supine-upright test showed hyperreninemia and hyperaldosteronism. Electrocardiogram, abdominal ultrasound, and adrenal computed tomography revealed no abnormalities.

**Table 1:**
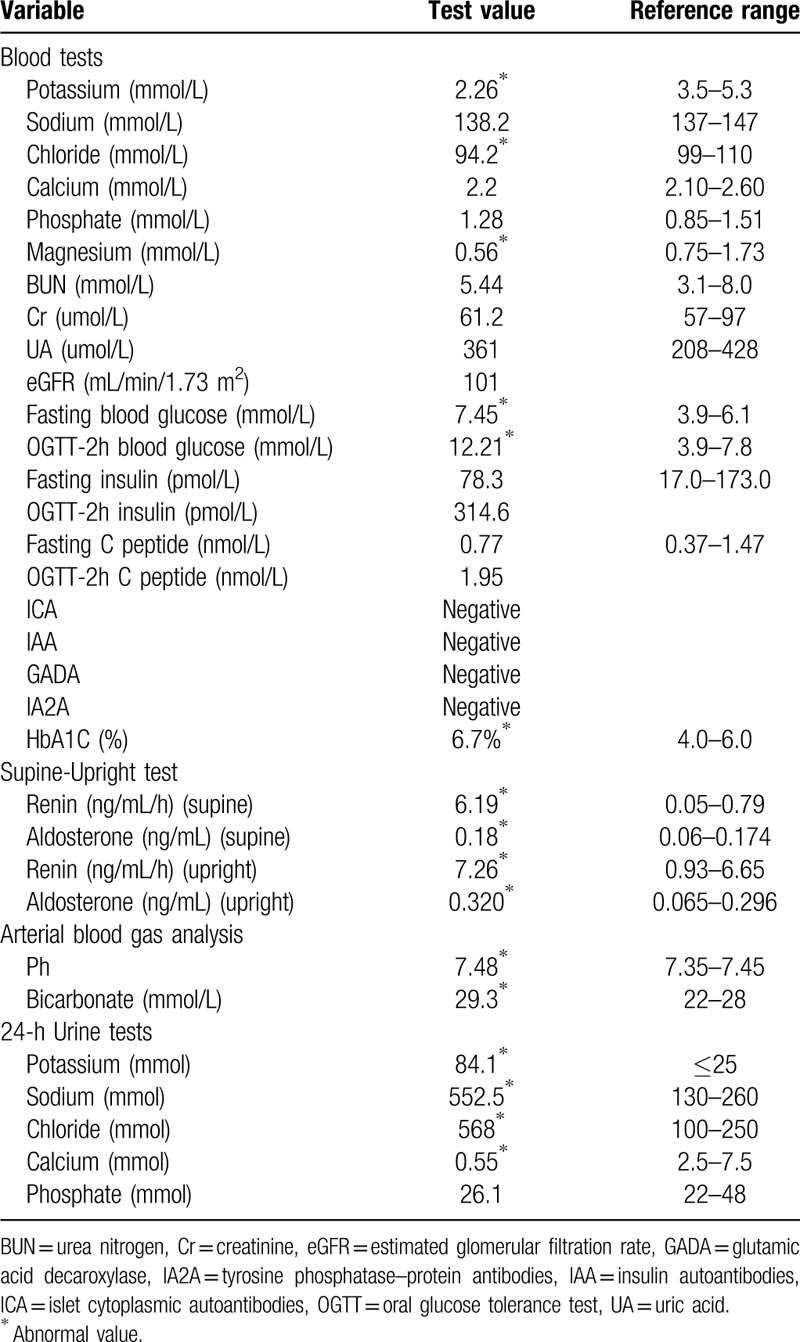
Laboratory tests of the proband.

In the current study, according to OGTT, islet function test, and diabetes antibody test results, the patient was diagnosed with T2DM. The criteria for T2DM diagnosis are high blood glucose (fasting plasma glucose ≥7.0 mmol/L, or 2 h-postprandial plasma glucose ≥11.1 mmol/L during OGTT, or glycosylated hemoglobin ≥6.5%, or random plasma glucose ≥11.1 mmol/L) with normal or elevated insulin levels; the diabetes-related antibodies are usually negative.^[9]^

**Figure 1:**
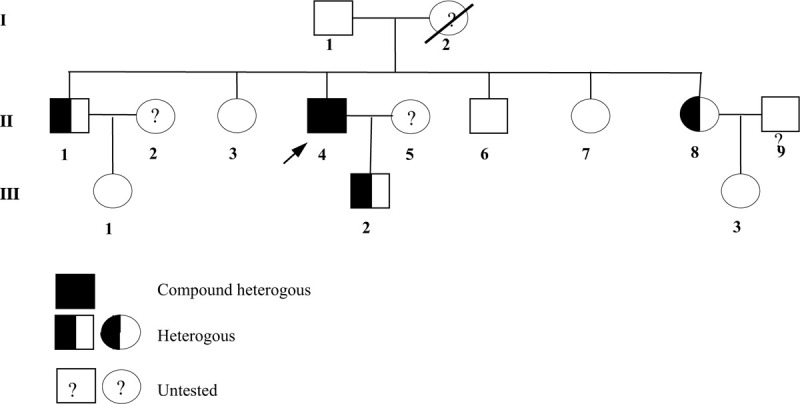
Pedigree chart of *SLC12A3* gene analysis. Men are indicated by squares, and women are indicated by circles. Proband is marked with an arrow.

**Figure 2:**
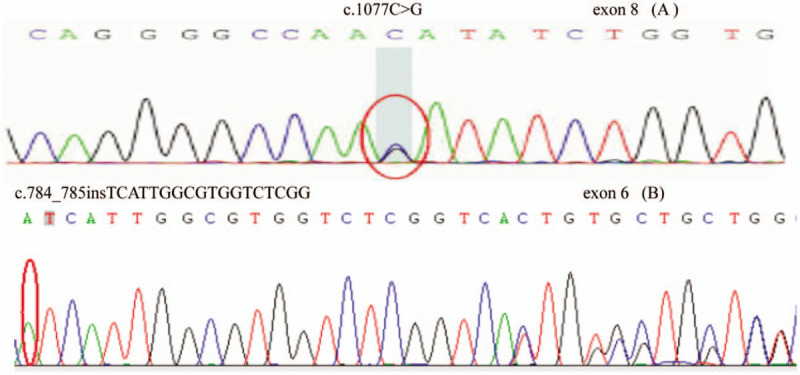
Genetic analysis of the *SLC12A3* gene. A, Missense mutation c.1077C>G in exon 8 leads to p.N359K. B, Novel insert mutation c.784_785insTCATTGGCGTGGTCTCGG in exon 6 leads to p.I262delinsIIGVVSV. The mutant nucleotides are encircled.

After obtaining written informed consent from the patient and his family, and the approval of the ethical review committee of The First Hospital of Jilin University (2016–397), the *SLC12A3* gene of the patient (Fig. 1, II4) was analyzed. The results showed 2 heterozygous mutations in the exon area of *SLC12A3* (Fig. 2): a missense mutation c.1077C>G in exon 8; the variant possessed lysine instead of asparagine at position 359 (p.N359K, NM_000339); this mutation has been reported as a pathogenic mutation for GS and a novel insert mutation c.784_785insTCATTGGCGTGGTCTCGG in exon 6, leading to p.I262delinsIIGVVSV. In addition, the *SLC12A3* genes of 9 other family members of the patient were analyzed, the missense mutation p.N359K was found in the older brother (Fig. 1, II1), youngest sister (Fig. 1, II8), and son of the patient (Fig. 1, III2).

**Figure 3:**
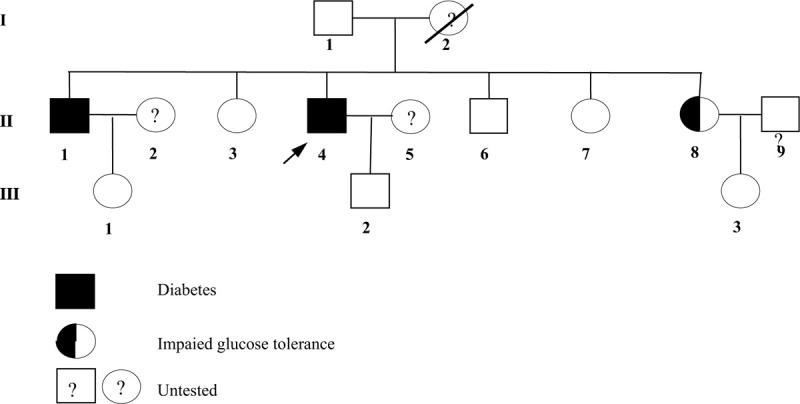
Pedigree chart of oral glucose tolerance test. Men are indicated by squares, and women are indicated by circles. Proband is marked with an arrow.

The OGTT results and blood potassium levels of the pedigree (Fig. 3) are summarized in Table 2. The patient's older brother (Fig. 3, II1) and youngest sister (Fig. 3, III8), who carried the pathogenic mutation, were diagnosed with T2DM and impaired glucose tolerance, respectively.

**Table 2:**
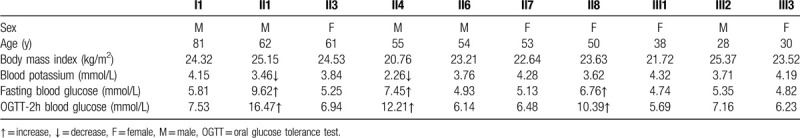
Demography, blood potassium, and oral glucose tolerance test results of pedigree.

Based on the typical symptoms, laboratory test results, and gene analysis, the patient was diagnosed with GS. The patient was prescribed oral supplements of potassium magnesium aspartate (316 mg/280 mg, 3 times per day), potassium chloride (500 mg, 3 times per day), and spironolactone (20 mg, 3 times per day). He was recommended to maintain a high potassium diet. The blood glucose levels were maintained using the drug acarbose (50 mg, 3 times per day). Blood potassium and blood magnesium levels fluctuated between 3.02 and 3.54 mmol/L, and 0.63 to 0.71 mmol/L, respectively. There were no adverse effects due to the drugs. Although acarbose was stopped after half year of the treatment, fasting plasma glucose levels and postprandial plasma glucose levels were still maintained at 5 to 6 mmol/L and 6 to 9 mmol/L, respectively. The patient was followed up for 2 years.

## 3 Discussion

Recently, it has been reported that patients with GS may be at an increased risk for developing T2DM,^[10,11]^ which is a type of diabetes due to a progressive loss of β-cell insulin secretion, frequently caused by insulin resistance. According to previous studies, 47.8% of Chinese GS patients have abnormal glucose metabolism and 19.4% have T2DM,^[12]^ while 4.3% of Taiwanese GS patients develop T2DM after an average follow-up of 11 years.^[13]^ Additionally, GS patients may have an earlier onset age of T2DM.^[14,15]^

Chronic hypokalemia and hypomagnesemia impair insulin secretion and insulin sensitivity^[14,15]^; and hyperaldosteronism increases insulin resistance^[16]^; the mechanisms by which they cause insulin secretion and sensitivity, and insulin resistance have been previously published. Thus, hypokalemia, hypomagnesemia, and hyperaldosteronism—the clinical manifestations of GS— could be responsible for occurrence of T2DM in GS patients. Hypokalemia prevents the closure of ATP-sensitive potassium channels on the β cell surface to prevent insulin secretion.^[17]^ This leads to β cell dysfunction and even apoptosis.^[18]^ Hypomagnesemia reduces tyrosine kinase activity at the insulin receptor level, and dysregulates K^+^-ATP and L-type Ca^2+^ channels in the β cells; thus, impairing insulin activity^[19]^ and reducing insulin secretion.^[20]^ Hyperaldosteronism can increase reactive oxygen species, and accelerate endothelial remodeling, which can reduce delivery of insulin for glucose metabolism.^[21]^ Excess aldosterone induces insulin resistance by reducing insulin receptor substrate-1 expression, and by blocking the downstream protein kinase B signaling in the vascular smooth muscles.^[22]^ Other studies have also shown that hyperaldosteronism induces insulin resistance in different tissues and organs, including adipose tissue^[23–25]^ and liver.^[26]^ Therefore, elevated blood potassium and magnesium levels, and reduced aldosterone level can improve impaired glucose tolerance. In the current study, the blood glucose levels of the patient improved after treatment with oral supplements of potassium and magnesium, and spironolactone.

Though sufficient research has not been done to describe the clinical features of GS patients with T2DM, it has been reported that T2DM coexisted with GS in some patients. However, there are only a few reports on cases wherein hyperglycemia did not occur before the diagnosis of GS or hypokalemia. Thus, in Table 3, we have summarized the details of GS patients from previous studies, including demography, genotype of GS, duration of hypokalemia, and onset age of both GS and T2DM.^[13,27,28]^ Based on the results, the mutations of GS patients who developed T2DM were observed to be more commonly located in the exon area (89%, 8/9) than in the intron area (21%, 1/9), including heterozygote (56%, 5/9) and homozygote (44%, 4/9). Male patients (78%, 7/9) were much more affected than the female patients (22%, 2/9). The development of T2DM in GS patients (78%, 7/9) seemed to be more prevalent in the Chinese people than in other races (32%, 2/9). It took an average duration of 18 years (18.22 ± 9.50 years) for GS patients to develop T2DM. However, the statistics are limited due to fewer studies on GS patients with T2DM. Therefore, large prospective studies on the relationship between GS and T2DM are needed.

**Table 3:**
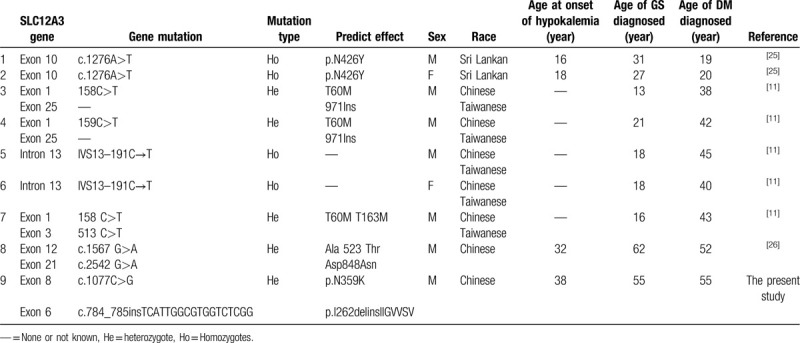
The characteristics of patients with Gitelman syndrome coexisted with type 2 diabetes.

A cross-sectional study of the patients with GS indicated that heterozygous carriers of *SLC12A3* variants had an intermediate phenotype between healthy noncarriers and GS patients.^[29]^ The mutation p.N359K of *SLC12A3* may be associated with impaired glucose metabolism (with or without hypokalemia), especially when the carriers are older, have an unhealthy lifestyle, and a higher BMI.^[29]^ Mutation p.N359K is not the only *SLC12A3* mutation associated with T2DM; mutation Arg913Gln of *SLC12A3* has also been known to predict the development and progression of end-stage renal disease in Chinese T2DM patients.^[30]^

Additionally, 18% to 40% of the suspected GS patients carry only 1 *SLC12A3* mutant allele,^[31]^ and heterozygous carriers are more susceptible to diuretic-induced hypokalemia.^[32]^ The family members with mutation p.N359K should monitor blood potassium regularly, and avoid diuretics.

## 4 Conclusion

Therefore, tests for diagnosis of GS should be considered when the patients present persistent, unexplained, and poorly controlled hypokalemia. Genetic analysis is a golden standard for the diagnosis of GS, and the target genetic analysis of pedigree may be more meaningful. Long-term treatment to maintain electrolyte balance, prevent complications, and improve prognosis poses a challenge for clinicians. In addition, GS patients, especially Chinese males with mutations in the exon area of the *SLC12A3* gene, should monitor blood glucose levels regularly. As it takes a long time for GS patients to develop T2DM, early glucose tolerance test and regular glucose monitoring are necessary.

Hyperaldosteronism, hypokalemia, and hypomagnesemia could be the main causes of T2DM induced by GS; hence, blood potassium, magnesium, and aldosterone levels should be controlled timely to prevent impaired glucose metabolism. Additionally, the siblings of GS patients who carry the *SLC12A3* mutation should also monitor their blood glucose and potassium levels. More studies are required to further investigate the relationship between GS and T2DM.

## Acknowledgments

The authors thank their patient and appreciate the help of all family members who participated in this study.

## Author contributions

**Conceptualization:** Guang-yu He, Xiao-Kun Gang.

**Data curation:** Guang-yu He, Zhonghua Sun.

**Formal analysis:** Zhonghua Sun.

**Funding acquisition:** Weiying Guo.

**Investigation:** Guixia Wang.

**Methodology:** Xiao-Kun Gang, Ping Wang.

**Supervision:** Guixia Wang, Weiying Guo.

**Writing – original draft:** Guang-yu He.

**Writing – review & editing:** Guang-yu He, Xiao-Kun Gang, Zhonghua Sun, Ping Wang, Guixia Wang, Weiying Guo.
